# What to Do When *K*-Means Clustering Fails: A Simple yet Principled Alternative Algorithm

**DOI:** 10.1371/journal.pone.0162259

**Published:** 2016-09-26

**Authors:** Yordan P. Raykov, Alexis Boukouvalas, Fahd Baig, Max A. Little

**Affiliations:** 1 School of Mathematics, Aston University, Birmingham, United Kingdom; 2 Molecular Sciences, University of Manchester, Manchester, United Kingdom; 3 Nuffield Department of Clinical Neurosciences, Oxford University, Oxford, United Kingdom; 4 Media Lab, Massachusetts Institute of Technology, Cambridge, Massachusetts, United States of America; Texas A&M University College Station, UNITED STATES

## Abstract

The *K*-means algorithm is one of the most popular clustering algorithms in current use as it is relatively fast yet simple to understand and deploy in practice. Nevertheless, its use entails certain restrictive assumptions about the data, the negative consequences of which are not always immediately apparent, as we demonstrate. While more flexible algorithms have been developed, their widespread use has been hindered by their computational and technical complexity. Motivated by these considerations, we present a flexible alternative to *K*-means that relaxes most of the assumptions, whilst remaining almost as fast and simple. This novel algorithm which we call MAP-DP (*maximum a-posteriori Dirichlet process mixtures*), is statistically rigorous as it is based on nonparametric Bayesian Dirichlet process mixture modeling. This approach allows us to overcome most of the limitations imposed by *K*-means. The number of clusters *K* is estimated from the data instead of being fixed *a-priori* as in *K*-means. In addition, while *K*-means is restricted to continuous data, the MAP-DP framework can be applied to many kinds of data, for example, binary, count or ordinal data. Also, it can efficiently separate outliers from the data. This additional flexibility does not incur a significant computational overhead compared to *K*-means with MAP-DP convergence typically achieved in the order of seconds for many practical problems. Finally, in contrast to *K*-means, since the algorithm is based on an underlying statistical model, the MAP-DP framework can deal with missing data and enables model testing such as cross validation in a principled way. We demonstrate the simplicity and effectiveness of this algorithm on the health informatics problem of clinical sub-typing in a cluster of diseases known as parkinsonism.

## 1 Introduction

The rapid increase in the capability of automatic data acquisition and storage is providing a striking potential for innovation in science and technology. However, extracting meaningful information from complex, ever-growing data sources poses new challenges. This motivates the development of automated ways to discover underlying structure in data. The key information of interest is often obscured behind redundancy and noise, and grouping the data into clusters with similar features is one way of efficiently summarizing the data for further analysis [1]. Cluster analysis has been used in many fields [1, 2], such as information retrieval [3], social media analysis [4], neuroscience [5], image processing [6], text analysis [7] and bioinformatics [8].

Despite the large variety of flexible models and algorithms for clustering available, *K*-means remains the preferred tool for most real world applications [9]. *K*-means was first introduced as a method for *vector quantization* in communication technology applications [10], yet it is still one of the most widely-used clustering algorithms. For example, in discovering *sub-types* of parkinsonism, we observe that most studies have used *K*-means algorithm to find sub-types in patient data [11]. It is also the preferred choice in the *visual bag of words* models in automated image understanding [12]. Perhaps the major reasons for the popularity of *K*-means are *conceptual simplicity* and *computational scalability*, in contrast to more flexible clustering methods. Bayesian probabilistic models, for instance, require complex *sampling schedules* or *variational inference* algorithms that can be difficult to implement and understand, and are often not computationally tractable for large data sets.

For the ensuing discussion, we will use the following mathematical notation to describe *K*-means clustering, and then also to introduce our novel clustering algorithm. Let us denote the data as *X* = (*x*_1_, …, *x*_*N*_) where each of the *N* data points *x*_*i*_ is a *D*-dimensional vector. We will denote the *cluster assignment* associated to each data point by *z*_1_, …, *z*_*N*_, where if data point *x*_*i*_ belongs to cluster *k* we write *z*_*i*_ = *k*. The number of observations assigned to cluster *k*, for *k* ∈ 1, …, *K*, is *N*_*k*_ and $N_{k}^{-i}$ is the number of points assigned to cluster *k* excluding point *i*. The parameter *ϵ* > 0 is a small threshold value to assess when the algorithm has converged on a good solution and should be stopped (typically *ϵ* = 10^−6^). Using this notation, *K*-means can be written as in Algorithm 1.

To paraphrase this algorithm: it alternates between updating the assignments of data points to clusters while holding the estimated cluster *centroids*, *μ*_*k*_, fixed (lines 5-11), and updating the cluster centroids while holding the assignments fixed (lines 14-15). It can be shown to find *some* minimum (not necessarily the *global*, i.e. smallest of all possible minima) of the following *objective function*:

$$E=\frac{1}{2}\overset{K}{\underset{k=1}{\sum }}\underset{i:z_{i}=k}{\sum }||x_{i}-\mu_{k}{\left|\right|}_{2}^{2}$$ (1)

with respect to the set of all cluster assignments *z* and cluster centroids *μ*, where $\begin{array}{c} \frac{1}{2}||.|{|}_{2}^{2} \end{array}$ denotes the *Euclidean distance* (distance measured as the sum of the square of differences of coordinates in each direction). In fact, the value of *E* *cannot increase* on each iteration, so, eventually *E* will stop changing (tested on line 17).

Perhaps unsurprisingly, the simplicity and computational scalability of *K*-means comes at a high cost. In particular, the algorithm is based on quite restrictive assumptions about the data, often leading to severe limitations in accuracy and interpretability:

**Table 1:** 

	Algorithm 1: *K*-means		Algorithm 2: MAP-DP(spherical Gaussian)
**Input**	*x*_1_, …, *x*_*N*_: *D*-dimensional data *ϵ* > 0: convergence threshold *K*: number of clusters		*x*_1_, …, *x*_*N*_: *D*-dimensional data *ϵ* > 0: convergence threshold *N*_0_: prior count ${\hat{\sigma }}^{2}$: spherical cluster variance $\sigma_{0}^{2}$: prior centroid variance *μ*_0_: prior centroid location
**Output**	*z*_1_, …, *z*_*N*_: cluster assignments *μ*_1_, …, *μ*_*K*_: cluster centroids		*z*_1_, …, *z*_*N*_: cluster assignments *K*: number of clusters
**1**	Set *μ*_*k*_ for all *k* ∈ 1, …, *K*	**1**	*K* = 1, *z*_*i*_ = 1 for all *i* ∈ 1, …, *N*
**2**	*E*_new_ = ∞	**2**	*E*_new_ = ∞
**3**	**repeat**	**3**	**repeat**
**4**	*E*_old_ = *E*_new_	**4**	*E*_old_ = *E*_new_
**5**	**for** *i* ∈ 1, …, *N*	**5**	**for** *i* ∈ 1, …, *N*
**6**	**for** *k* ∈ 1, …, *K*	**6**	**for** *k* ∈ 1, …, *K*
**7**		**7**	$\sigma_{k}^{-i}={\left(\frac{1}{\sigma_{0}^{2}}+\frac{1}{{\hat{\sigma }}^{2}}N_{k}^{-i}\right)}^{-1}$
**8**		**8**	$\mu_{k}^{-i}=\sigma_{k}^{-i}(\frac{\mu_{0}}{\sigma_{0}^{2}}+\frac{1}{{\hat{\sigma }}^{2}}\sum_{j:z_{j}=k,j\neq i}x_{j})$
**9**	$d_{i,k}=\frac{1}{2}||x_{i}-\mu_{k}|{|}_{2}^{2}$	**9**	$d_{i,k}=\frac{1}{2\left(\sigma_{k}^{-i}+{\hat{\sigma }}^{2}\right)}||x_{i}-\mu_{k}^{-i}|{|}_{2}^{2}+\frac{D}{2}\text{ln}\left(\sigma_{k}^{-i}+{\hat{\sigma }}^{2}\right)$
**10**		**10**	$d_{i,K+1}=\frac{1}{2\left(\sigma_{0}^{2}+{\hat{\sigma }}^{2}\right)}||x_{i}-\mu_{0}|{|}_{2}^{2}+\frac{D}{2}\text{ln}\left(\sigma_{0}^{2}+{\hat{\sigma }}^{2}\right)$
**11**	$z_{i}={arg min}_{k\in 1,\ldots ,K}d_{i,k}$	**11**	$z_{i}={arg min}_{k\in 1,\ldots ,K+1}[d_{i,k}-\text{ln}N_{k}^{-i}]$
**12**		**12**	**if** *z*_*i*_ = *K* + 1
**13**		**13**	*K* = *K* + 1
**14**	**for** *k* ∈ 1, …, *K*	**14**	
**15**	$\mu_{k}=\frac{1}{N_{k}}\sum_{j:z_{j}=k}x_{j}$	**15**	
**16**	$E_{\text{new}}=\sum_{k=1}^{K}\sum_{i:z_{i}=k}d_{i,k}$	**16**	$E_{new}=\sum \begin{array}{l} K\\ k=1 \end{array}\sum \begin{array}{l} i:z_{i}=k \end{array}d_{i,k}-K\text{ln}N_{0}-\overset{K}{\underset{k=1}{\sum }}\text{log}\Gamma \left(N_{k}\right)$
**17**	**until** *E*_old_ − *E*_new_ < *ϵ*	**17**	**until** *E*_old_ − *E*_new_ < *ϵ*

**Fig 1:**
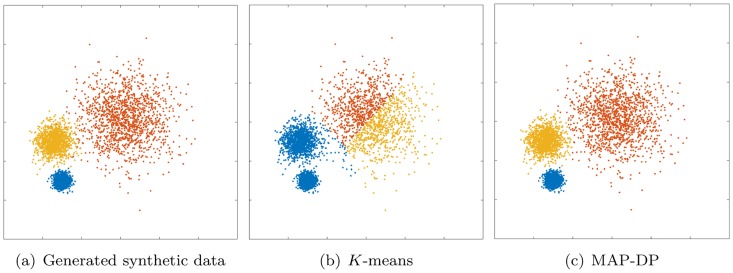
Clustering performed by *K*-means and MAP-DP for spherical, synthetic Gaussian data, with unequal cluster radii and density. The clusters are well-separated. Data is equally distributed across clusters. Here, unlike MAP-DP, *K*-means fails to find the correct clustering. Instead, it splits the data into three equal-volume regions because it is insensitive to the differing cluster density. Different colours indicate the different clusters.

**Fig 2:**
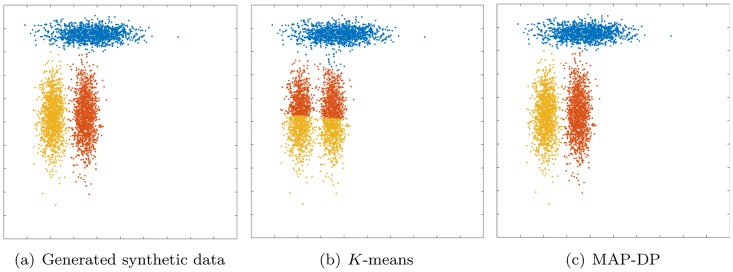
Clustering solution obtained by *K*-means and MAP-DP for synthetic elliptical Gaussian data. All clusters share exactly the same volume and density, but one is rotated relative to the others. There is no appreciable overlap. *K*-means fails because the objective function which it attempts to minimize measures the true clustering solution as worse than the manifestly poor solution shown here.

**Fig 3:**
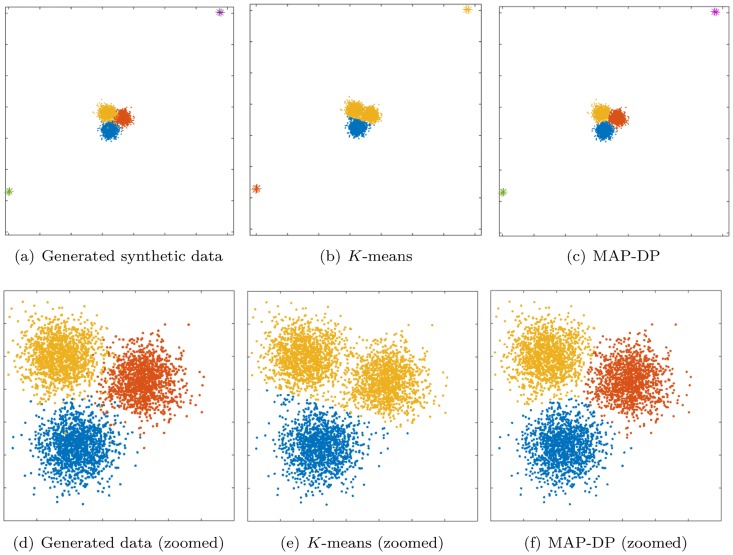
Clustering performed by *K*-means and MAP-DP for spherical, synthetic Gaussian data, with outliers. All clusters have the same radii and density. There are two outlier groups with two outliers in each group. *K*-means fails to find a good solution where MAP-DP succeeds; this is because *K*-means puts some of the outliers in a separate cluster, thus inappropriately using up one of the *K* = 3 clusters. This happens even if all the clusters are spherical, equal radii and well-separated.

**Fig 4:**
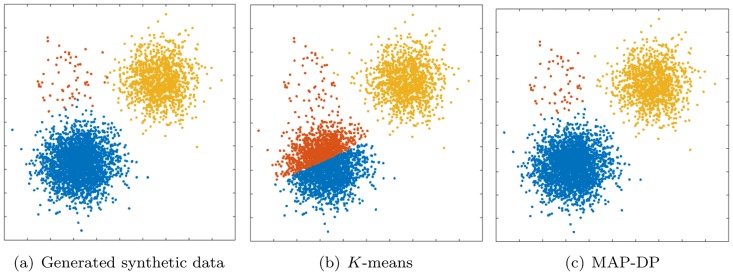
Clustering performed by *K*-means and MAP-DP for spherical, synthetic Gaussian data. Cluster radii are equal and clusters are well-separated, but the data is unequally distributed across clusters: 69% of the data is in the blue cluster, 29% in the yellow, 2% is orange. *K*-means fails to find a meaningful solution, because, unlike MAP-DP, it cannot adapt to different cluster densities, even when the clusters are spherical, have equal radii and are well-separated.

1. By use of the Euclidean distance (algorithm line 9) *K*-means treats the data space as *isotropic* (distances unchanged by translations and rotations). This means that data points in each cluster are modeled as lying within a *sphere* around the cluster centroid. A sphere has the same radius in each dimension. Furthermore, as clusters are modeled only by the position of their centroids, *K*-means implicitly assumes all clusters have the same radius. When this implicit equal-radius, spherical assumption is violated, *K*-means can behave in a non-intuitive way, even when clusters are very clearly identifiable by eye (see Figs 1 and 2 and discussion in Sections 5.1, 5.4).
2. The Euclidean distance entails that the average of the coordinates of data points in a cluster is the centroid of that cluster (algorithm line 15). Euclidean space is *linear* which implies that small changes in the data result in proportionately small changes to the position of the cluster centroid. This is problematic when there are *outliers*, that is, points which are unusually far away from the cluster centroid by comparison to the rest of the points in that cluster. Such outliers can dramatically impair the results of *K*-means (see Fig 3 and discussion in Section 5.3).
3. *K*-means clusters data points purely on their (Euclidean) *geometric closeness* to the cluster centroid (algorithm line 9). Therefore, it does not take into account the different *densities* of each cluster. So, because *K*-means implicitly assumes each cluster occupies the same volume in data space, each cluster must contain the same number of data points. We will show later that even when all other implicit geometric assumptions of *K*-means are satisfied, it will fail to learn a correct, or even meaningful, clustering when there are significant differences in cluster density (see Fig 4 and Section 5.2).
4. The number *K* of groupings in the data is fixed and assumed known; this is rarely the case in practice. Thus, *K*-means is quite inflexible and degrades badly when the assumptions upon which it is based are even mildly violated by e.g. a tiny number of outliers (see Fig 3 and discussion in Section 5.3).

Some of the above limitations of *K*-means have been addressed in the literature. Regarding outliers, variations of *K*-means have been proposed that use more “robust” estimates for the cluster centroids. For example, the *K-medoids* algorithm uses the point in each cluster which is most centrally located. By contrast, in *K-medians* the median of coordinates of all data points in a cluster is the centroid. However, both approaches are far more computationally costly than *K*-means. *K*-medoids, requires computation of a pairwise similarity matrix between data points which can be prohibitively expensive for large data sets. In *K*-medians, the coordinates of cluster data points in each dimension need to be sorted, which takes much more effort than computing the mean.

Provided that a transformation of the entire data space can be found which “spherizes” each cluster, then the spherical limitation of *K*-means can be mitigated. However, for most situations, finding such a transformation will not be trivial and is usually as difficult as finding the clustering solution itself. Alternatively, by using the *Mahalanobis distance*, *K*-means can be adapted to non-spherical clusters [13], but this approach will encounter problematic computational singularities when a cluster has only one data point assigned.

Addressing the problem of the fixed number of clusters *K*, note that it is not possible to choose *K* simply by clustering with a range of values of *K* and choosing the one which minimizes *E*. This is because *K*-means is *nested*: we can always decrease *E* by increasing *K*, even when the true number of clusters is much smaller than *K*, since, all other things being equal, *K*-means tries to create an equal-volume partition of the data space. Therefore, data points find themselves ever closer to a cluster centroid as *K* increases. In the extreme case for *K* = *N* (the number of data points), then *K*-means will assign each data point to its own separate cluster and *E* = 0, which has no meaning as a “clustering” of the data. Various extensions to *K*-means have been proposed which circumvent this problem by *regularization* over *K*, e.g. *Akaike*(AIC) or *Bayesian information criteria* (BIC), and we discuss this in more depth in Section 3).

So far, we have presented *K*-means from a geometric viewpoint. However, it can also be profitably understood from a probabilistic viewpoint, as a restricted case of the (*finite*) *Gaussian mixture model* (GMM). This is the starting point for us to introduce a new algorithm which overcomes most of the limitations of *K*-means described above.

This new algorithm, which we call *maximum a-posteriori Dirichlet process* mixtures (MAP-DP), is a more flexible alternative to *K*-means which can quickly provide interpretable clustering solutions for a wide array of applications.

By contrast to *K*-means, MAP-DP can perform cluster analysis without specifying the number of clusters. In order to model *K* we turn to a probabilistic framework where *K* grows with the data size, also known as *Bayesian non-parametric*(BNP) models [14]. In particular, we use *Dirichlet process mixture models*(DP mixtures) where the number of clusters can be estimated from data. To date, despite their considerable power, applications of DP mixtures are somewhat limited due to the computationally expensive and technically challenging inference involved [15, 16, 17]. Our new MAP-DP algorithm is a computationally scalable and simple way of performing inference in DP mixtures. Additionally, MAP-DP is model-based and so provides a consistent way of inferring missing values from the data and making predictions for unknown data.

As a prelude to a description of the MAP-DP algorithm in full generality later in the paper, we introduce a special (simplified) case, Algorithm 2, which illustrates the key similarities and differences to *K*-means (for the case of spherical Gaussian data with known cluster variance; in Section 4 we will present the MAP-DP algorithm in full generality, removing this spherical restriction):

- The number of clusters *K* is not fixed but inferred from the data. The algorithm is initialized with *K* = 1 and all data points assigned to one cluster (MAP-DP algorithm line 1). In the assignment step (algorithm line 11), a choice is made between assigning the current data point to one of the existing clusters (algorithm line 9) or assigning it to a *prior cluster* located at *μ*_0_ with variance $\sigma_{0}^{2}$ (algorithm line 10). When $\sigma_{k}^{-i}\approx \sigma_{0}^{2}$ and the current data point is the same distance from *μ*_0_ and from the current most likely cluster centroid $\mu_{k}^{-i}$, a new cluster is created (algorithm lines 12, 13) only if the *prior count* (*concentration*) parameter $N_{0}>N_{k}^{-i}$. In other words, all other things being geometrically similar, only the *relative counts* of the number of data points in each cluster, and the prior count, determines whether a new cluster is created or not. By contrast, if $\sigma_{k}^{-i}$ is very different from $\sigma_{0}^{2}$, then the geometry largely determines the creation of new clusters: if a data point is closer to the prior location *μ*_0_ than to any other most likely existing cluster centroid, $\mu_{k}^{-i}$, then a new cluster is created.
- In this spherical variant of MAP-DP, as with *K*-means, the Euclidean metric $\begin{array}{c} \frac{1}{2}||.|{|}_{2}^{2} \end{array}$ is used to compute distances to cluster centroids (algorithm lines 9, 10). However, in MAP-DP, the log of $N_{k}^{-i}$ is subtracted from this distance when updating assignments (algorithm line 11). Also, the composite variance $\sigma_{k}^{-i}+{\hat{\sigma }}^{2}$ features in the distance calculations such that the smaller $\sigma_{k}^{-i}+{\hat{\sigma }}^{2}$ becomes, the less important the number of data points in the cluster $N_{k}^{-i}$ becomes to the assignment. In that case, the algorithm behaves much like *K*-means. But, if $\sigma_{k}^{-i}+{\hat{\sigma }}^{2}$ becomes large, then, if a cluster already has many data points assigned to it, it is more likely that the current data point is assigned to that cluster (in other words, clusters exhibit a “*rich-get-richer*” effect). MAP-DP thereby takes into account the density of clusters, unlike *K*-means. We can see $\sigma_{k}^{-i}+{\hat{\sigma }}^{2}$ as controlling the “balance” between geometry and density.
- MAP-DP directly estimates only cluster assignments, while *K*-means also finds the most likely cluster centroids given the current cluster assignments. But, since the cluster assignment estimates may be significantly in error, this error will propagate to the most likely cluster centroid locations. By contrast, MAP-DP never explicitly estimates cluster centroids, they are treated as appropriately uncertain quantities described by a most likely cluster location $\mu_{k}^{-i}$ and variance $\sigma_{k}^{-i}$ (the centroid *hyper parameters*). This means that MAP-DP does not need explicit values of the cluster centroids on initialization (*K*-means algorithm line 1). Indeed, with *K*-means, poor choices of these initial cluster centroids can cause the algorithm to fall into sub-optimal configurations from which it cannot recover, and there is, generally, no known universal way to pick “good” initial centroids. At the same time, during iterations of the algorithm, MAP-DP can bypass sub-optimal, erroneous configurations that *K*-means cannot avoid. This also means that MAP-DP often converges in many fewer iterations than *K*-means. As we discuss in Appendix C cluster centroids and variances can be obtained in MAP-DP if needed after the algorithm has converged.
- The cluster hyper parameters are updated explicitly for each data point in turn (algorithm lines 7, 8). This updating is a *weighted sum* of *prior location* *μ*_0_ and the mean of the data currently assigned to each cluster. If the *prior variance* parameter $\sigma_{0}^{2}$ is large or the known cluster variance ${\hat{\sigma }}^{2}$ is small, then *μ*_*k*_ is just the mean of the data in cluster *k*, as with *K*-means. By contrast, if the prior variance is small (or the known cluster variance ${\hat{\sigma }}^{2}$ is large), then *μ*_*k*_ ≈ *μ*_0_, the prior centroid location. So, intuitively, the most likely location of the cluster centroid is based on an appropriate “balance” between the confidence we have in the data in each cluster and our prior information about the cluster centroid location.
- While *K*-means estimates only the cluster centroids, this spherical Gaussian variant of MAP-DP has an additional cluster variance parameter ${\hat{\sigma }}^{2}$, effectively determining the radius of the clusters. If the prior variance $\sigma_{0}^{2}$ or the cluster variance ${\hat{\sigma }}^{2}$ are small, then $\sigma_{k}^{-i}$ becomes small. This is the situation where we have high confidence in the most likely cluster centroid *μ*_*k*_. If, on the other hand, the prior variance $\sigma_{0}^{2}$ is large, then $\sigma_{k}^{-i}\approx \frac{{\hat{\sigma }}^{2}}{N_{k}^{-i}}$. Intuitively, if we have little trust in the prior location *μ*_0_, the more data in each cluster, the better the estimate of the most likely cluster centroid. Finally, for large cluster variance ${\hat{\sigma }}^{2}$, then $\sigma_{k}^{-i}\approx \sigma_{0}^{2}$, so that the uncertainty in the most likely cluster centroid defaults to that of the prior.

A summary of the paper is as follows. In Section 2 we review the *K*-means algorithm and its derivation as a constrained case of a GMM. Section 3 covers alternative ways of choosing the number of clusters. In Section 4 the novel MAP-DP clustering algorithm is presented, and the performance of this new algorithm is evaluated in Section 5 on synthetic data. In Section 6 we apply MAP-DP to explore phenotyping of parkinsonism, and we conclude in Section 8 with a summary of our findings and a discussion of limitations and future directions.

## 2 A probabilistic interpretation of *K*-means

In order to improve on the limitations of *K*-means, we will invoke an interpretation which views it as an inference method for a specific kind of *mixture model*. While *K*-means is essentially geometric, mixture models are inherently *probabilistic*, that is, they involve fitting a probability density model to the data. The advantage of considering this probabilistic framework is that it provides a *mathematically* *principled* way to understand and address the limitations of *K*-means. It is well known that *K*-means can be derived as an approximate inference procedure for a special kind of finite mixture model. For completeness, we will rehearse the derivation here.

### 2.1 Finite mixture models

In the GMM (p. 430-439 in [18]) we assume that data points are drawn from a *mixture* (a weighted sum) of Gaussian distributions with density $p(x)=\sum_{k=1}^{K}\pi_{k}N(x|\mu_{k},\Sigma_{k})$, where *K* is the fixed number of components, *π*_*k*_ > 0 are the weighting coefficients with $\sum_{k=1}^{K}\pi_{k}=1$, and *μ*_*k*_, *Σ*_*k*_ are the parameters of each Gaussian in the mixture. So, to produce a data point *x*_*i*_, the model first draws a cluster assignment *z*_*i*_ = *k*. The distribution over each *z*_*i*_ is known as a *categorical distribution* with *K* parameters *π*_*k*_ = *p*(*z*_*i*_ = *k*). Then, given this assignment, the data point is drawn from a Gaussian with mean *μ*_*z*_*i*__ and covariance *Σ*_*z*_*i*__.

Under this model, the conditional probability of each data point is $p(x_{i}|z_{i}=k)=N(x_{i}|\mu_{k},\Sigma_{k})$, which is just a Gaussian. But an equally important quantity is the probability we get by reversing this conditioning: the probability of an assignment *z*_*i*_ given a data point *x* (sometimes called the *responsibility*), *p*(*z*_*i*_ = *k*|*x*, *μ*_*k*_, *Σ*_*k*_). This raises an important point: in the GMM, a data point has a finite probability of belonging to *every* cluster, whereas, for *K*-means each point belongs to only one cluster. This is because the GMM is *not* a partition of the data: the assignments *z*_*i*_ are treated as random draws from a distribution.

One of the most popular algorithms for estimating the unknowns of a GMM from some data (that is the variables *z*, *μ*, *Σ* and *π*) is the *Expectation-Maximization* (E-M) algorithm. This iterative procedure alternates between the *E* (*expectation*) step and the *M* (*maximization*) steps. The E-step uses the responsibilities to compute the cluster assignments, holding the cluster parameters fixed, and the M-step re-computes the cluster parameters holding the cluster assignments fixed:

**E-step:** Given the current estimates for the cluster parameters, compute the responsibilities:

$$\begin{array}{c} \gamma_{i,k}=p\left(z_{i}=k\left|x,\mu_{k},\Sigma_{k}\right.\right)=\frac{\pi_{k}N\left(x_{i}\left|\mu_{k},\Sigma_{k}\right.\right)}{\sum_{j=1}^{K}\pi_{j}N\left(x_{i}\left|\mu_{j},\Sigma_{j}\right.\right)} \end{array}$$ (2)

**M-step:** Compute the parameters that maximize the *likelihood* of the data set *p*(*X*|*π*, *μ*, *Σ*, *z*), which is the probability of all of the data under the GMM [19]:

$$\begin{array}{c} p\left(X\left|\pi ,\mu ,\Sigma ,z\right.\right)=\prod_{i=1}^{N}\sum_{k=1}^{K}\pi_{k}N\left(x_{i}\left|\mu_{k},\Sigma_{k}\right.\right) \end{array}$$ (3)

Maximizing this with respect to each of the parameters can be done in closed form:

$$\begin{array}{c} \begin{array}{cc} S_{k}=\sum_{i=1}^{N}\gamma_{i,k} & \pi_{k}=\frac{S_{k}}{N}\\ \mu_{k}=\frac{1}{S_{k}}\sum_{i=1}^{N}\gamma_{i,k}x_{i} & \Sigma_{k}=\frac{1}{S_{k}}\sum_{i=1}^{N}\gamma_{i,k}\left(x_{i}-\mu_{k}\right){\left(x_{i}-\mu_{k}\right)}^{T} \end{array} \end{array}$$ (4)

Each E-M iteration is guaranteed not to decrease the likelihood function *p*(*X*|*π*, *μ*, *Σ*, *z*). So, as with *K*-means, convergence is guaranteed, but not necessarily to the global maximum of the likelihood. We can, alternatively, say that the E-M algorithm attempts to minimize the GMM objective function:

$$\begin{array}{c} E=-\sum_{i=1}^{N}\text{ln}\sum_{k=1}^{K}\pi_{k}N\left(x_{i}\left|\mu_{k},\Sigma_{k}\right.\right) \end{array}$$ (5)

When changes in the likelihood are sufficiently small the iteration is stopped.

### 2.2 Connection to *K*-means

We can derive the *K*-means algorithm from E-M inference in the GMM model discussed above. Consider a special case of a GMM where the covariance matrices of the mixture components are spherical and shared across components. That means *Σ*_*k*_ = *σI* for *k* = 1, …, *K*, where *I* is the *D* × *D* identity matrix, with the variance *σ* > 0. We will also assume that *σ* is a known constant. Then the E-step above simplifies to:

$$\begin{array}{c} \gamma_{i,k}=\frac{\pi_{k}\exp\left(-\frac{1}{2\sigma }{||x_{i}-\mu_{k}||}_{2}^{2}\right)}{\sum_{j=1}^{K}\pi_{j}\exp\left(-\frac{1}{2\sigma }{||x_{i}-\mu_{j}||}_{2}^{2}\right)} \end{array}$$ (6)

The M-step no longer updates the values for *Σ*_*k*_ at each iteration, but otherwise it remains unchanged.

Now, let us further consider shrinking the constant variance term to 0: *σ* → 0. At this limit, the responsibility probability Eq (6) takes the value 1 for the component which is closest to *x*_*i*_. That is, of course, the component for which the (squared) Euclidean distance $\frac{1}{2}||x_{i}-\mu_{k}|{|}_{2}^{2}$ is minimal. So, all other components have responsibility 0. Also at the limit, the categorical probabilities *π*_*k*_ cease to have any influence. In effect, the E-step of E-M behaves exactly as the assignment step of *K*-means. Similarly, since *π*_*k*_ has no effect, the M-step re-estimates only the mean parameters *μ*_*k*_, which is now just the sample mean of the data which is closest to that component.

To summarize, if we assume a probabilistic GMM model for the data with fixed, identical spherical covariance matrices across all clusters and take the limit of the cluster variances *σ* → 0, the E-M algorithm becomes equivalent to *K*-means. This has, more recently, become known as the *small variance asymptotic* (SVA) derivation of *K*-means clustering [20].

## 3 Inferring *K*, the number of clusters

The GMM (Section 2.1) and mixture models in their full generality, are a principled approach to modeling the data beyond purely geometrical considerations. As such, mixture models are useful in overcoming the equal-radius, equal-density spherical cluster limitation of *K*-means. Nevertheless, it still leaves us empty-handed on choosing *K* as in the GMM this is a fixed quantity.

The choice of *K* is a well-studied problem and many approaches have been proposed to address it. As discussed above, the *K*-means objective function Eq (1) cannot be used to select *K* as it will always favor the larger number of components. Probably the most popular approach is to run *K*-means with different values of *K* and use a regularization principle to pick the best *K*. For instance in Pelleg and Moore [21], BIC is used. Bischof et al. [22] use *minimum description length*(MDL) regularization, starting with a value of *K* which is larger than the expected true value for *K* in the given application, and then removes centroids until changes in description length are minimal. By contrast, Hamerly and Elkan [23] suggest starting *K*-means with one cluster and splitting clusters until points in each cluster have a Gaussian distribution. An obvious limitation of this approach would be that the Gaussian distributions for each cluster need to be spherical. In Gao et al. [24] the choice of *K* is explored in detail leading to the *deviance information criterion* (DIC) as regularizer. DIC is most convenient in the probabilistic framework as it can be readily computed using *Markov chain Monte Carlo* (MCMC). In addition, DIC can be seen as a hierarchical generalization of BIC and AIC.

All these regularization schemes consider ranges of values of *K* and must perform exhaustive restarts for each value of *K*. This increases the computational burden. By contrast, our MAP-DP algorithm is based on a model in which the number of clusters is just another random variable in the model (such as the assignments *z*_*i*_). So, *K* is estimated as an intrinsic part of the algorithm in a more computationally efficient way.

As argued above, the likelihood function in GMM Eq (3) and the sum of Euclidean distances in *K*-means Eq (1) cannot be used to compare the fit of models for different *K*, because this is an ill-posed problem that cannot detect overfitting. A natural way to regularize the GMM is to assume priors over the uncertain quantities in the model, in other words to turn to *Bayesian models*. Placing priors over the cluster parameters smooths out the cluster shape and penalizes models that are too far away from the expected structure [25]. Also, placing a prior over the cluster weights provides more control over the distribution of the cluster densities. The key in dealing with the uncertainty about *K* is in the prior distribution we use for the cluster weights *π*_*k*_, as we will show.

In MAP-DP, instead of fixing the number of components, we will assume that the more data we observe the more clusters we will encounter. For many applications this is a reasonable assumption; for example, if our aim is to extract different variations of a disease given some measurements for each patient, the expectation is that with more patient records more subtypes of the disease would be observed. As another example, when extracting topics from a set of documents, as the number and length of the documents increases, the number of topics is also expected to increase. When clustering similar companies to construct an efficient financial portfolio, it is reasonable to assume that the more companies are included in the portfolio, a larger variety of company clusters would occur.

Formally, this is obtained by assuming that *K* → ∞ as *N* → ∞, but with *K* growing more slowly than *N* to provide a meaningful clustering. But, for any finite set of data points, the number of clusters is always some unknown but finite *K*^+^ that can be inferred from the data. The parametrization of *K* is avoided and instead the model is controlled by a new parameter *N*_0_ called the *concentration parameter* or *prior count*. This controls the rate with which *K* grows with respect to *N*. Additionally, because there is a consistent probabilistic model, *N*_0_ may be estimated from the data by standard methods such as maximum likelihood and cross-validation as we discuss in Appendix F.

## 4 Generalized MAP-DP algorithm

Before presenting the model underlying MAP-DP (Section 4.2) and detailed algorithm (Section 4.3), we give an overview of a key probabilistic structure known as the *Chinese restaurant process*(CRP). The latter forms the theoretical basis of our approach allowing the treatment of *K* as an unbounded random variable.

### 4.1 The Chinese restaurant process (CRP)

In clustering, the essential discrete, combinatorial structure is a *partition* of the data set into a finite number of groups, *K*. The CRP is a probability distribution on these partitions, and it is parametrized by the prior count parameter *N*_0_ and the number of data points *N*. For a partition example, let us assume we have data set *X* = (*x*_1_, …, *x*_*N*_) of just *N* = 8 data points, one particular partition of this data is the set {{*x*_1_, *x*_2_}, {*x*_3_, *x*_5_, *x*_7_}, {*x*_4_, *x*_6_}, {*x*_8_}}. In this partition there are *K* = 4 clusters and the cluster assignments take the values *z*_1_ = *z*_2_ = 1, *z*_3_ = *z*_5_ = *z*_7_ = 2, *z*_4_ = *z*_6_ = 3 and *z*_8_ = 4. So, we can also think of the CRP as a distribution over cluster assignments.

The CRP is often described using the metaphor of a restaurant, with data points corresponding to customers and clusters corresponding to tables. Customers arrive at the restaurant one at a time. The first customer is seated alone. Each subsequent customer is either seated at one of the already occupied tables with probability proportional to the number of customers already seated there, or, with probability proportional to the parameter *N*_0_, the customer sits at a new table. We use *k* to denote a cluster index and *N*_*k*_ to denote the number of customers sitting at table *k*. With this notation, we can write the probabilistic rule characterizing the CRP:

$$\begin{array}{c} p\left(\text{customer}\;i+1\;\text{joins}\;\text{table}\;k\right)=\left\{\begin{array}{cc} \frac{N_{k}}{N_{0}+i} & \text{if}\;k\;\text{is}\;\text{an}\;\text{existing}\;\text{table}\\ \frac{N_{0}}{N_{0}+i} & \text{if}\;k\;\text{is}\;\text{a}\;\text{new}\;\text{table} \end{array}\right. \end{array}$$ (7)

After *N* customers have arrived and so *i* has increased from 1 to *N*, their seating pattern defines a set of clusters that have the CRP distribution. This partition is random, and thus the CRP is a distribution on partitions and we will denote a draw from this distribution as:

$$\begin{array}{c} \left(z_{1},\ldots ,z_{N}\right)∼\text{CRP}\left(N_{0},N\right) \end{array}$$ (8)

Further, we can compute the probability over all cluster assignment variables, given that they are a draw from a CRP:

$$\begin{array}{c} p\left(z_{1},\ldots ,z_{N}\right)=\frac{N_{0}^{K}}{N_{0}^{\left(N\right)}}\prod_{k=1}^{K}\left(N_{k}-1\right)! \end{array}$$ (9)

where $N_{0}^{\left(N\right)}=N_{0}\left(N_{0}+1\right)\times \cdots \times \left(N_{0}+N-1\right)$. This probability is obtained from a product of the probabilities in Eq (7). If there are exactly *K* tables, customers have sat on a new table exactly *K* times, explaining the term $N_{0}^{K}$ in the expression. The probability of a customer sitting on an existing table *k* has been used *N*_*k*_ − 1 times where each time the numerator of the corresponding probability has been increasing, from 1 to *N*_*k*_ − 1. This is how the term $\prod_{k=1}^{K}(N_{k}-1)!$ arises. The $N_{0}^{\left(N\right)}$ is the product of the denominators when multiplying the probabilities from Eq (7), as *N* = 1 at the start and increases to *N* − 1 for the last seated customer.

Notice that the CRP is *solely* parametrized by the number of customers (data points) *N* and the concentration parameter *N*_0_ that controls the probability of a customer sitting at a new, unlabeled table. We can see that the parameter *N*_0_ controls the rate of increase of the number of tables in the restaurant as *N* increases. It is usually referred to as the concentration parameter because it controls the typical density of customers seated at tables.

We can think of there being an infinite number of unlabeled tables in the restaurant at any given point in time, and when a customer is assigned to a new table, one of the unlabeled ones is chosen arbitrarily and given a numerical label. We can think of the number of unlabeled tables as *K*, where *K* → ∞ and the number of labeled tables would be some random, but finite *K*^+^ < *K* that could increase each time a new customer arrives.

### 4.2 The underlying probabilistic model

First, we will model the distribution over the cluster assignments *z*_1_, …, *z*_*N*_ with a CRP (in fact, we can derive the CRP from the assumption that the mixture weights *π*_1_, …, *π*_*K*_ of the finite mixture model, Section 2.1, have a *DP prior*; see Teh [26] for a detailed exposition of this fascinating and important connection). We will also place priors over the other random quantities in the model, the cluster parameters. We will restrict ourselves to assuming *conjugate priors* for computational simplicity (however, this assumption is not essential and there is extensive literature on using non-conjugate priors in this context [16, 27, 28]).

As we are mainly interested in clustering applications, i.e. we are only interested in the cluster assignments *z*_1_, …, *z*_*N*_, we can gain computational efficiency [29] by *integrating out* the cluster parameters (this process of eliminating random variables in the model which are not of explicit interest is known as *Rao-Blackwellization* [30]). The resulting probabilistic model, called the *CRP mixture model* by Gershman and Blei [31], is:

$$\begin{array}{c} \begin{array}{ccc} \left(z_{1},\ldots ,z_{N}\right) & ∼ & \text{CRP}\left(N_{0},N\right)\\ x_{i} & ∼ & f\left(\theta_{z_{i}}\right) \end{array} \end{array}$$ (10)

where *θ* are the hyper parameters of the *predictive distribution* *f*(*x*|*θ*). Detailed expressions for this model for some different data types and distributions are given in (S1 Material). To summarize: we will assume that data is described by some random *K*^+^ number of predictive distributions describing each cluster where the randomness of *K*^+^ is parametrized by *N*_0_, and *K*^+^ increases with *N*, at a rate controlled by *N*_0_.

### 4.3 MAP-DP algorithm

Much as *K*-means can be derived from the more general GMM, we will derive our novel clustering algorithm based on the model Eq (10) above. The likelihood of the data *X* is:

$$\begin{array}{c} \begin{array}{cc} p\left(X,z|N_{0}\right)= & p\left(z_{1},\ldots ,z_{N}\right)\prod_{i=1}^{N}\prod_{k=1}^{K}f\left(x_{i}|\theta_{k}^{-i}\right){}^{\delta \left(z_{i},k\right)} \end{array} \end{array}$$ (11)

where *δ*(*x*, *y*) = 1 if *x* = *y* and 0 otherwise. The distribution *p*(*z*_1_, …, *z*_*N*_) is the CRP Eq (9). For ease of subsequent computations, we use the negative log of Eq (11):

$$\begin{array}{c} E=-\sum_{k=1}^{K}\sum_{i:z_{i}=k}\text{ln }f\left(x_{i}|\theta_{k}^{-i}\right)-K\;\text{ln}N_{0}-\sum_{k=1}^{K}\text{ln}\Gamma \left(N_{k}\right)-C\left(N_{0},N\right) \end{array}$$ (12)

where $C(N_{0},N)=\text{ln}\frac{\Gamma (N_{0})}{\Gamma (N_{0}+N)}$ is a function which depends upon only *N*_0_ and *N*. This can be omitted in the MAP-DP algorithm because it does not change over iterations of the main loop but should be included when estimating *N*_0_ using the methods proposed in Appendix F. The quantity Eq (12) plays an analogous role to the objective function Eq (1) in *K*-means. We wish to maximize Eq (11) over the only remaining random quantity in this model: the cluster assignments *z*_1_, …, *z*_*N*_, which is equivalent to minimizing Eq (12) with respect to *z*. This minimization is performed iteratively by optimizing over each cluster indicator *z*_*i*_, holding the rest, *z*_*j*:*j*≠*i*_, fixed. This is our MAP-DP algorithm, described in Algorithm 3 below.

For each data point *x*_*i*_, given *z*_*i*_ = *k*, we first update the posterior cluster hyper parameters $\theta_{k}^{-i}$ based on all data points assigned to cluster *k*, but excluding the data point *x*_*i*_ [16]. This update allows us to compute the following quantities for each existing cluster *k* ∈ 1, … *K*, and for a new cluster *K* + 1:

$$\begin{array}{c} \begin{array}{ccc} d_{i,k} & = & -\text{ln}\;f\left(x_{i}|\theta_{k}^{-i}\right)\\ d_{i,K+1} & = & -\text{ln}\;f\left(x_{i}|\theta_{0}\right) \end{array} \end{array}$$ (13)

Now, the quantity $d_{i,k}-\text{ln }N_{k}^{-i}$ is the negative log of the probability of assigning data point *x*_*i*_ to cluster *k*, or if we abuse notation somewhat and define $N_{K+1}^{-i}\equiv N_{0}$, assigning instead to a new cluster *K* + 1. Therefore, the MAP assignment for *x*_*i*_ is obtained by computing $z_{i}=\arg\min_{k\in 1,\ldots ,,K+1}[d_{i,k}-\text{ln }N_{k}^{-i}]$. Then the algorithm moves on to the next data point *x*_*i*+1_. Detailed expressions for different data types and corresponding predictive distributions *f* are given in (S1 Material), including the spherical Gaussian case given in Algorithm 2.

The objective function Eq (12) is used to assess convergence, and when changes between successive iterations are smaller than *ϵ*, the algorithm terminates. MAP-DP is guaranteed not to increase Eq (12) at each iteration and therefore the algorithm will converge [25]. By contrast to SVA-based algorithms, the closed form likelihood Eq (11) can be used to estimate hyper parameters, such as the concentration parameter *N*_0_ (see Appendix F), and can be used to make predictions for new *x* data (see Appendix D). In contrast to *K*-means, there exists a well founded, model-based way to infer *K* from data.

We summarize all the steps in Algorithm 3. The issue of randomisation and how it can enhance the robustness of the algorithm is discussed in Appendix B. During the execution of both K-means and MAP-DP empty clusters may be allocated and this can effect the computational performance of the algorithms; we discuss this issue in Appendix A.

For multivariate data a particularly simple form for the predictive density is to assume independent features. This means that the predictive distributions *f*(*x*|*θ*) over the data will factor into products with *M* terms, $f(x|\theta )=\prod_{m=1}^{M}f(x^{m}|\theta^{m})$ where *x*^*m*^, *θ*^*m*^ denotes the data and parameter vector for the *m*-th feature respectively. We term this the elliptical model. Including different types of data such as counts and real numbers is particularly simple in this model as there is no dependency between features. We demonstrate its utility in Section 6 where a multitude of data types is modeled.

**Table 2:** 

	Algorithm 3: MAP-DP (generalized algorithm)
**Input**	*x*_1_, …, *x*_*N*_: data *ϵ* > 0: convergence threshold *N*_0_: prior count *θ*_0_: prior hyper parameters
**Output**	*z*_1_, …, *z*_*N*_: cluster assignments *K*: number of clusters
**1**	*K* = 1, *z*_*i*_ = 1 for all *i* ∈ 1, …, *N*
**2**	*E*_new_ = ∞
**3**	**repeat**
**4**	*E*_old_ = *E*_new_
**5**	**for** *i* ∈ 1, …, *N*
**6**	**for** *k* ∈ 1, …, *K*
**7**	Update cluster hyper parameters $\theta_{k}^{-i}$ (see (S1 Material))
**8**	$d_{i,k}=-\text{ln }f(x_{i}|\theta_{k}^{-i})$
**9**	*d*_*i*,*K*+1_ = −ln *f*(*x*_*i*_|*θ*_0_)
**10**	$z_{i}={arg min}_{k\in 1,\ldots ,K+1}[d_{i,k}-\text{ln }N_{k}^{-i}]$
**11**	**if** *z*_*i*_ = *K* + 1
**12**	*K* = *K* + 1
**13**	$E_{\text{new}}=\sum_{k=1}^{K}\sum_{i:z_{i}=k}d_{i,k}-K\text{ ln }N_{0}-\sum_{k=1}^{K}\log\;\Gamma (N_{k})$
**14**	**until** *E*_old_ − *E*_new_ < *ϵ*

## 5 Study of synthetic data

In this section we evaluate the performance of the MAP-DP algorithm on six different synthetic Gaussian data sets with *N* = 4000 points. All these experiments use multivariate normal distribution with multivariate Student-t predictive distributions *f*(*x*|*θ*) (see (S1 Material)). The data sets have been generated to demonstrate some of the non-obvious problems with the *K*-means algorithm. Comparisons between MAP-DP, *K*-means, E-M and the Gibbs sampler demonstrate the ability of MAP-DP to overcome those issues with minimal computational and conceptual “overhead”. Both the E-M algorithm and the Gibbs sampler can also be used to overcome most of those challenges, however both aim to estimate the posterior density rather than clustering the data and so require significantly more computational effort.

The true clustering assignments are known so that the performance of the different algorithms can be objectively assessed. For the purpose of illustration we have generated two-dimensional data with three, visually separable clusters, to highlight the specific problems that arise with *K*-means. To ensure that the results are stable and reproducible, we have performed multiple restarts for *K*-means, MAP-DP and E-M to avoid falling into obviously sub-optimal solutions. MAP-DP restarts involve a random permutation of the ordering of the data.

*K*-means and E-M are restarted with randomized parameter initializations. Note that the initialization in MAP-DP is trivial as all points are just assigned to a single cluster, furthermore, the clustering output is less sensitive to this type of initialization. At the same time, *K*-means and the E-M algorithm require setting initial values for the cluster centroids *μ*_1_, …, *μ*_*K*_, the number of clusters *K* and in the case of E-M, values for the cluster covariances *Σ*_1_, …, *Σ*_*K*_ and cluster weights *π*_1_, …, *π*_*K*_. The clustering output is quite sensitive to this initialization: for the *K*-means algorithm we have used the seeding heuristic suggested in [32] for initialiazing the centroids (also known as the *K*-means++ algorithm); herein the E-M has been given an advantage and is initialized with the true generating parameters leading to quicker convergence. In all of the synthethic experiments, we fix the prior count to *N*_0_ = 3 for both MAP-DP and Gibbs sampler and the prior hyper parameters *θ*_0_ are evaluated using *empirical bayes* (see Appendix F).

To evaluate algorithm performance we have used *normalized mutual information* (NMI) between the true and estimated partition of the data (Table 3). The NMI between two random variables is a measure of mutual dependence between them that takes values between 0 and 1 where the higher score means stronger dependence. NMI scores close to 1 indicate good agreement between the estimated and true clustering of the data.

**Table 3:** Comparing the clustering performance of MAP-DP (multivariate normal variant), *K*-means, E-M and Gibbs sampler in terms of NMI which has range [0, 1] on synthetic Gaussian data generated using a GMM with *K* = 3. NMI closer to 1 indicates better clustering.

Geometry	Shared geometry?	Shared population?	Section	NMI *K*-means	NMI MAP-DP	NMI E-M	NMI Gibbs
Spherical	No	Yes	5.1	0.57	0.97	0.89	0.92
Spherical	Yes	No	5.2	0.48	0.98	0.98	0.86
Spherical	Yes	Yes	5.3	0.67	0.93	0.65	0.91
Elliptical	No	Yes	5.4	0.56	0.98	0.93	0.90
Elliptical	No	No	5.5	1.00	1.00	0.99	1.00
Elliptical	No	No	5.6	0.56	0.88	0.86	0.84

We also test the ability of regularization methods discussed in Section 3 to lead to sensible conclusions about the underlying number of clusters *K* in *K*-means. We use the BIC as a representative and popular approach from this class of methods. For all of the data sets in Sections 5.1 to 5.6, we vary *K* between 1 and 20 and repeat *K*-means 100 times with randomized initializations. That is, we estimate BIC score for *K*-means at convergence for *K* = 1, …, 20 and repeat this cycle 100 times to avoid conclusions based on sub-optimal clustering results. The theory of BIC suggests that, on each cycle, the value of *K* between 1 and 20 that maximizes the BIC score is the optimal *K* for the algorithm under test. We report the value of *K* that maximizes the BIC score over all cycles.

We also report the number of iterations to convergence of each algorithm in Table 4 as an indication of the relative computational cost involved, where the iterations include only a single run of the corresponding algorithm and ignore the number of restarts. The Gibbs sampler was run for 600 iterations for each of the data sets and we report the number of iterations until the draw from the chain that provides the best fit of the mixture model. Running the Gibbs sampler for a longer number of iterations is likely to improve the fit. Due to its stochastic nature, random restarts are not common practice for the Gibbs sampler.

**Table 4:** Number of iterations to convergence of MAP-DP, *K*-means, E-M and Gibbs sampling where one iteration consists of a full sweep through the data and the model parameters. The computational cost per iteration is not exactly the same for different algorithms, but it is comparable. The number of iterations due to randomized restarts have not been included.

Section	Convergence *K*-means	Convergence MAP-DP	Convergence E-M	Convergence Gibbs sampler 1
5.1	6	11	10	299
5.2	13	5	21	403
5.3	5	5	32	292
5.4	15	11	6	330
5.5	6	7	21	459
5.6	9	11	7	302

### 5.1 Spherical data, unequal cluster radius and density

In this example we generate data from three spherical Gaussian distributions with different radii. The data is well separated and there is an equal number of points in each cluster. In Fig 1 we can see that *K*-means separates the data into three almost *equal-volume* clusters. In *K*-means clustering, volume is not measured in terms of the density of clusters, but rather the geometric volumes defined by hyper-planes separating the clusters. The algorithm does not take into account cluster density, and as a result it splits large radius clusters and merges small radius ones. This would obviously lead to inaccurate conclusions about the structure in the data. It is unlikely that this kind of clustering behavior is desired in practice for this dataset. The poor performance of *K*-means in this situation reflected in a low NMI score (0.57, Table 3). By contrast, MAP-DP takes into account the density of each cluster and learns the true underlying clustering almost perfectly (NMI of 0.97). This shows that *K*-means can fail even when applied to spherical data, provided only that the cluster radii are different. Assuming the number of clusters *K* is unknown and using *K*-means with BIC, we can estimate the true number of clusters *K* = 3, but this involves defining a range of possible values for *K* and performing multiple restarts for each value in that range. Considering a range of values of *K* between 1 and 20 and performing 100 random restarts for each value of *K*, the estimated value for the number of clusters is *K* = 2, an underestimate of the true number of clusters *K* = 3. The highest BIC score occurred after 15 cycles of *K* between 1 and 20 and as a result, *K*-means with BIC required significantly longer run time than MAP-DP, to correctly estimate *K*.

### 5.2 Spherical data, equal cluster radius, unequal density

In this next example, data is generated from three spherical Gaussian distributions with equal radii, the clusters are well-separated, but with a different number of points in each cluster. In Fig 4 we observe that the most populated cluster containing 69% of the data is split by *K*-means, and a lot of its data is assigned to the smallest cluster. So, despite the unequal density of the true clusters, *K*-means divides the data into three almost equally-populated clusters. Again, this behaviour is non-intuitive: it is unlikely that the *K*-means clustering result here is what would be desired or expected, and indeed, *K*-means scores badly (NMI of 0.48) by comparison to MAP-DP which achieves near perfect clustering (NMI of 0.98. Table 3). The reason for this poor behaviour is that, if there is *any* overlap between clusters, *K*-means will attempt to resolve the ambiguity by dividing up the data space into equal-volume regions. This will happen even if all the clusters are spherical with equal radius. Again, assuming that *K* is unknown and attempting to estimate using BIC, after 100 runs of *K*-means across the whole range of *K*, we estimate that *K* = 2 maximizes the BIC score, again an underestimate of the true number of clusters *K* = 3.

### 5.3 Spherical data, equal cluster radius and density, with outliers

Next we consider data generated from three spherical Gaussian distributions with equal radii and equal density of data points. However, we add two pairs of outlier points, marked as stars in Fig 3. We see that *K*-means groups together the top right outliers into a cluster of their own. As a result, one of the pre-specified *K* = 3 clusters is wasted and there are only two clusters left to describe the actual spherical clusters. So, *K*-means merges two of the underlying clusters into one and gives misleading clustering for at least a third of the data. For this behavior of *K*-means to be avoided, we would need to have information not only about how many groups we would expect in the data, but also how many outlier points might occur. By contrast, since MAP-DP estimates *K*, it can adapt to the presence of outliers. MAP-DP assigns the two pairs of outliers into separate clusters to estimate *K* = 5 groups, and correctly clusters the remaining data into the three true spherical Gaussians. Again, *K*-means scores poorly (NMI of 0.67) compared to MAP-DP (NMI of 0.93, Table 3). From this it is clear that *K*-means is not “robust” to the presence of even a trivial number of outliers, which can severely degrade the quality of the clustering result. For many applications, it is infeasible to remove all of the outliers before clustering, particularly when the data is high-dimensional. If we assume that *K* is unknown for *K*-means and estimate it using the BIC score, we estimate *K* = 4, an overestimate of the true number of clusters *K* = 3. We further observe that even the E-M algorithm with Gaussian components does not handle outliers well and the nonparametric MAP-DP and Gibbs sampler are clearly the more robust option in such scenarios.

### 5.4 Elliptical data with equal cluster volumes and densities, rotated

So far, in all cases above the data is spherical. By contrast, we next turn to non-spherical, in fact, elliptical data. This next experiment demonstrates the inability of *K*-means to correctly cluster data which is trivially separable by eye, even when the clusters have negligible overlap and exactly equal volumes and densities, but simply because the data is non-spherical and some clusters are rotated relative to the others. Fig 2 shows that *K*-means produces a very misleading clustering in this situation. 100 random restarts of *K*-means fail to find any better clustering, with *K*-means scoring badly (NMI of 0.56) by comparison to MAP-DP (0.98, Table 3). In fact, for this data, we find that even if *K*-means is initialized with the *true* cluster assignments, this is not a fixed point of the algorithm and *K*-means will continue to degrade the true clustering and converge on the poor solution shown in Fig 2. So, this clustering solution obtained at *K*-means convergence, as measured by the objective function value *E* Eq (1), appears to actually be better (i.e. lower) than the true clustering of the data. Essentially, for some non-spherical data, the objective function which *K*-means attempts to minimize is fundamentally incorrect: even if *K*-means can find a small value of *E*, it is solving the wrong problem. Furthermore, BIC does not provide us with a sensible conclusion for the correct underlying number of clusters, as it estimates *K* = 9 after 100 randomized restarts.

It should be noted that in some rare, non-spherical cluster cases, global transformations of the entire data can be found to “spherize” it. For example, if the data is elliptical and all the cluster covariances are the same, then there is a global linear transformation which makes all the clusters spherical. However, finding such a transformation, if one exists, is likely at least as difficult as first correctly clustering the data.

### 5.5 Elliptical data with different cluster volumes, geometries and densities, no cluster overlap

**Fig 5:**
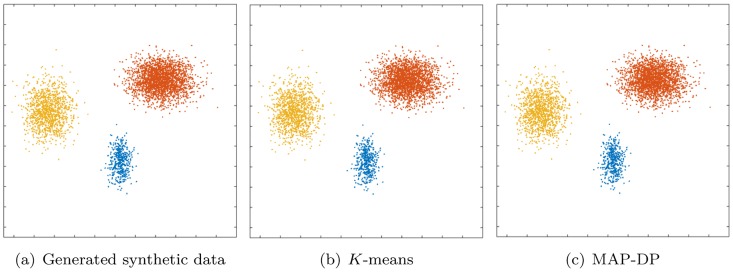
Clustering solution obtained by *K*-means and MAP-DP for synthetic elliptical Gaussian data. The clusters are trivially well-separated, and even though they have different densities (12% of the data is blue, 28% yellow cluster, 60% orange) and elliptical cluster geometries, *K*-means produces a near-perfect clustering, as with MAP-DP. This shows that *K*-means can in some instances work when the clusters are not equal radii with shared densities, but only when the clusters are so well-separated that the clustering can be trivially performed by eye.

This data is generated from three elliptical Gaussian distributions with different covariances and different number of points in each cluster. In this case, despite the clusters not being spherical, equal density and radius, the clusters are so well-separated that *K*-means, as with MAP-DP, can perfectly separate the data into the correct clustering solution (see Fig 5). So, for data which is trivially separable by eye, *K*-means can produce a meaningful result. However, it is questionable how often in practice one would expect the data to be so clearly separable, and indeed, whether computational cluster analysis is actually necessary in this case. Even in this trivial case, the value of *K* estimated using BIC is *K* = 4, an overestimate of the true number of clusters *K* = 3.

### 5.6 Elliptical data with different cluster volumes and densities, significant overlap

**Fig 6:**
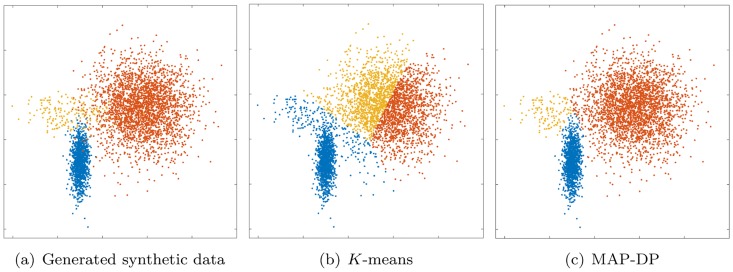
Clustering solution obtained by *K*-means and MAP-DP for overlapping, synthetic elliptical Gaussian data. All clusters have different elliptical covariances, and the data is unequally distributed across different clusters (30% blue cluster, 5% yellow cluster, 65% orange). The significant overlap is challenging even for MAP-DP, but it produces a meaningful clustering solution where the only mislabelled points lie in the overlapping region. *K*-means does not produce a clustering result which is faithful to the actual clustering.

Having seen that MAP-DP works well in cases where *K*-means can fail badly, we will examine a clustering problem which should be a challenge for MAP-DP. The data is generated from three elliptical Gaussian distributions with different covariances and different number of points in each cluster. There is significant overlap between the clusters. MAP-DP manages to correctly learn the number of clusters in the data and obtains a good, meaningful solution which is close to the truth (Fig 6, NMI score 0.88, Table 3). The small number of data points mislabeled by MAP-DP are all in the overlapping region. By contrast, *K*-means fails to perform a meaningful clustering (NMI score 0.56) and mislabels a large fraction of the data points that are outside the overlapping region. This shows that MAP-DP, unlike *K*-means, can easily accommodate departures from sphericity even in the context of significant cluster overlap. As the cluster overlap increases, MAP-DP degrades but always leads to a much more interpretable solution than *K*-means. In this example, the number of clusters can be correctly estimated using BIC.

## 6 Example application: sub-typing of parkinsonism and Parkinson’s disease

*Parkinsonism* is the clinical syndrome defined by the combination of bradykinesia (slowness of movement) with tremor, rigidity or postural instability. This clinical syndrome is most commonly caused by *Parkinson’s disease*(PD), although can be caused by drugs or other conditions such as multi-system atrophy. Because of the common clinical features shared by these other causes of parkinsonism, the clinical diagnosis of PD in vivo is only 90% accurate when compared to post-mortem studies. This diagnostic difficulty is compounded by the fact that PD itself is a heterogeneous condition with a wide variety of clinical phenotypes, likely driven by different disease processes. These include wide variations in both the *motor* (movement, such as tremor and gait) and *non-motor* symptoms (such as cognition and sleep disorders). While the motor symptoms are more specific to parkinsonism, many of the non-motor symptoms associated with PD are common in older patients which makes clustering these symptoms more complex. Despite significant advances, the aetiology (underlying cause) and pathogenesis (how the disease develops) of this disease remain poorly understood, and no disease modifying treatment has yet been found.

The diagnosis of PD is therefore likely to be given to some patients with other causes of their symptoms. Also, even with the correct diagnosis of PD, they are likely to be affected by different disease mechanisms which may vary in their response to treatments, thus reducing the power of clinical trials. Despite numerous attempts to classify PD into sub-types using empirical or data-driven approaches (using mainly *K*-means cluster analysis), there is no widely accepted consensus on classification.

One approach to identifying PD and its subtypes would be through appropriate clustering techniques applied to comprehensive data sets representing many of the physiological, genetic and behavioral features of patients with parkinsonism. We expect that a clustering technique should be able to identify PD subtypes as distinct from other conditions. In that context, using methods like *K*-means and finite mixture models would severely limit our analysis as we would need to fix a-priori the number of sub-types *K* for which we are looking. Estimating that *K* is still an open question in PD research. Potentially, the number of sub-types is not even fixed, instead, with increasing amounts of clinical data on patients being collected, we might expect a growing number of variants of the disease to be observed. A natural probabilistic model which incorporates that assumption is the DP mixture model. Here we make use of MAP-DP clustering as a computationally convenient alternative to fitting the DP mixture.

We have analyzed the data for 527 patients from the *PD data and organizing center* (PD-DOC) clinical reference database, which was developed to facilitate the planning, study design, and statistical analysis of PD-related data [33]. The subjects consisted of patients referred with suspected parkinsonism thought to be caused by PD. Each patient was rated by a specialist on a percentage probability of having PD, with 90-100% considered as probable PD (this variable was not included in the analysis). This data was collected by several independent clinical centers in the US, and organized by the University of Rochester, NY. Ethical approval was obtained by the independent ethical review boards of each of the participating centres. From that database, we use the PostCEPT data.

For each patient with parkinsonism there is a comprehensive set of features collected through various questionnaires and clinical tests, in total 215 features per patient. The features are of different types such as yes/no questions, finite ordinal numerical rating scales, and others, each of which can be appropriately modeled by e.g. Bernoulli (yes/no), binomial (ordinal), categorical (nominal) and Poisson (count) random variables (see (S1 Material)). For simplicity and interpretability, we assume the different features are independent and use the elliptical model defined in Section 4.

A common problem that arises in health informatics is missing data. When using *K*-means this problem is usually separately addressed prior to clustering by some type of *imputation* method. However, in the MAP-DP framework, we can simultaneously address the problems of clustering and missing data. In the CRP mixture model Eq (10) the missing values are treated as an additional set of random variables and MAP-DP proceeds by updating them at every iteration. As a result, the missing values and cluster assignments will depend upon each other so that they are consistent with the observed feature data and each other.

We initialized MAP-DP with 10 randomized permutations of the data and iterated to convergence on each randomized restart. The results (Tables 5 and 6) suggest that the PostCEPT data is clustered into 5 groups with 50%, 43%, 5%, 1.6% and 0.4% of the data in each cluster. We then performed a Student’s t-test at *α* = 0.01 significance level to identify features that differ significantly between clusters. As with most hypothesis tests, we should always be cautious when drawing conclusions, particularly considering that not all of the mathematical assumptions underlying the hypothesis test have necessarily been met. Nevertheless, this analysis suggest that there are 61 features that differ significantly between the two largest clusters. Note that if, for example, none of the features were significantly different between clusters, this would call into question the extent to which the clustering is meaningful at all. We assume that the features differing the most among clusters are the same features that lead the patient data to cluster. By contrast, features that have indistinguishable distributions across the different groups should not have significant influence on the clustering.

**Table 5:** Significant features of parkinsonism from the PostCEPT/PD-DOC clinical reference data across clusters (groups) obtained using MAP-DP with appropriate distributional models for each feature. Each entry in the table is the probability of PostCEPT parkinsonism patient answering “yes” in each cluster (group).

	Group 1	Group 2	Group 3	Group 4
Resting tremor (present and typical)	0.81	0.91	0.42	0.78
Resting tremor (absent)	0.14	0.06	0.42	0.11
Symptoms in the past week	0.58	0.94	1.00	0.67

**Table 6:** Significant features of parkinsonism from the PostCEPT/PD-DOC clinical reference data across clusters obtained using MAP-DP with appropriate distributional models for each feature. Each entry in the table is the mean score of the ordinal data in each row. Lower numbers denote condition closer to healthy. Note that the Hoehn and Yahr stage is re-mapped from {0, 1.0, 1.5, 2, 2.5, 3, 4, 5} to {0, 1, 2, 3, 4, 5, 6, 7} respectively.

Mean score	Scale	Group1	Group 2	Group 3	Group 4
Facial expression	0-4	1.42	1.47	0.42	2.33
Tremor at rest (face, lips and chin)	0-4	0.05	0.32	0.23	1.00
Rigidity (right upper extremity)	0-4	0.90	1.30	0.38	2.11
Rigidity (left upper extremity)	0-4	0.62	1.33	0.19	2.00
Rigidity (right lower extremity)	0-4	0.46	0.97	0.04	2.56
Rigidity (left lower extremity)	0-4	0.38	1.06	0.04	2.67
Finger taps (left hand)	0-4	0.65	1.41	0.50	2.33
PD state during exam	1-4	2.65	3.85	4.00	3.00
Modified Hoehn and Yahr stage	0-7	2.46	3.19	1.62	6.33

We applied the significance test to each pair of clusters excluding the smallest one as it consists of only 2 patients. Exploring the full set of multilevel correlations occurring between 215 features among 4 groups would be a challenging task that would change the focus of this work. We therefore concentrate only on the pairwise-significant features between Groups 1-4, since the hypothesis test has higher power when comparing larger groups of data. The clustering results suggest many other features not reported here that differ significantly between the different pairs of clusters that could be further explored. Individual analysis on Group 5 shows that it consists of 2 patients with advanced parkinsonism but are unlikely to have PD itself (both were thought to have <50% probability of having PD).

Due to the nature of the study and the fact that very little is yet known about the sub-typing of PD, direct numerical validation of the results is not feasible. The purpose of the study is to learn in a completely unsupervised way, an interpretable clustering on this comprehensive set of patient data, and then interpret the resulting clustering by reference to other sub-typing studies.

Our analysis successfully clustered almost all the patients thought to have PD into the 2 largest groups. Only 4 out of 490 patients (which were thought to have Lewy-body dementia, multi-system atrophy and essential tremor) were included in these 2 groups, each of which had phenotypes very similar to PD. Because the unselected population of parkinsonism included a number of patients with phenotypes very different to PD, it may be that the analysis was therefore unable to distinguish the subtle differences in these cases. The fact that a few cases were not included in these group could be due to: an extreme phenotype of the condition; variance in how subjects filled in the self-rated questionnaires (either comparatively under or over stating symptoms); or that these patients were misclassified by the clinician. The inclusion of patients thought not to have PD in these two groups could also be explained by the above reasons.

Comparing the two groups of PD patients (Groups 1 & 2), group 1 appears to have less severe symptoms across most motor and non-motor measures. Group 2 is consistent with a more aggressive or rapidly progressive form of PD, with a lower ratio of tremor to rigidity symptoms. van Rooden et al. [11] combined the conclusions of some of the most prominent, large-scale studies. Of these studies, 5 distinguished rigidity-dominant and tremor-dominant profiles [34, 35, 36, 37]. Pathological correlation provides further evidence of a difference in disease mechanism between these two phenotypes. Our analysis, identifies a two subtype solution most consistent with a less severe tremor dominant group and more severe non-tremor dominant group most consistent with Gasparoli et al. [37].

These results demonstrate that even with small datasets that are common in studies on parkinsonism and PD sub-typing, MAP-DP is a useful exploratory tool for obtaining insights into the structure of the data and to formulate useful hypothesis for further research.

Although the clinical heterogeneity of PD is well recognized across studies [38], comparison of clinical sub-types is a challenging task. Studies often concentrate on a limited range of more specific clinical features. For instance, some studies concentrate only on cognitive features or on motor-disorder symptoms [5]. In addition, typically the cluster analysis is performed with the *K*-means algorithm and fixing *K* a-priori might seriously distort the analysis.

It is important to note that the clinical data itself in PD (and other neurodegenerative diseases) has inherent inconsistencies between individual cases which make sub-typing by these methods difficult: the clinical diagnosis of PD is only 90% accurate; medication causes inconsistent variations in the symptoms; clinical assessments (both self rated and clinician administered) are subjective; delayed diagnosis and the (variable) slow progression of the disease makes disease duration inconsistent. Therefore, any kind of partitioning of the data has inherent limitations in how it can be interpreted with respect to the known PD disease process. It may therefore be more appropriate to use the fully statistical DP mixture model to find the distribution of the joint data instead of focusing on the modal point estimates for each cluster. Our analysis presented here has the additional layer of complexity due to the inclusion of patients with parkinsonism without a clinical diagnosis of PD. This makes differentiating further subtypes of PD more difficult as these are likely to be far more subtle than the differences between the different causes of parkinsonism.

## 7 Limitations and extensions

Despite the broad applicability of the *K*-means and MAP-DP algorithms, their simplicity limits their use in some more complex clustering tasks. When facing such problems, devising a more application-specific approach that incorporates additional information about the data may be essential. For example, in cases of high dimensional data (*M* > > *N*) neither *K*-means, nor MAP-DP are likely to be appropriate clustering choices. Methods have been proposed that specifically handle such problems, such as a family of Gaussian mixture models that can efficiently handle high dimensional data [39]. Since MAP-DP is derived from the nonparametric mixture model, by incorporating subspace methods into the MAP-DP mechanism, an efficient high-dimensional clustering approach can be derived using MAP-DP as a building block. We leave the detailed exposition of such extensions to MAP-DP for future work.

Another issue that may arise is where the data cannot be described by an exponential family distribution. Clustering such data would involve some additional approximations and steps to extend the MAP approach. Fortunately, the exponential family is a rather rich set of distributions and is often flexible enough to achieve reasonable performance even where the data cannot be exactly described by an exponential family distribution.

We may also wish to cluster sequential data. In this scenario *hidden Markov models* [40] have been a popular choice to replace the simpler mixture model, in this case the MAP approach can be extended to incorporate the additional time-ordering assumptions [41].

## 8 Conclusion

This paper has outlined the major problems faced when doing clustering with *K*-means, by looking at it as a restricted version of the more general finite mixture model. We have presented a less restrictive procedure that retains the key properties of an underlying probabilistic model, which itself is more flexible than the finite mixture model. Making use of Bayesian nonparametrics, the new MAP-DP algorithm allows us to learn the number of clusters in the data and model more flexible cluster geometries than the spherical, Euclidean geometry of *K*-means. Additionally, it gives us tools to deal with missing data and to make predictions about new data points outside the training data set. At the same time, by avoiding the need for sampling and variational schemes, the complexity required to find good parameter estimates is almost as low as *K*-means with few conceptual changes. Like *K*-means, MAP-DP iteratively updates assignments of data points to clusters, but the distance in data space can be more flexible than the Euclidean distance. Unlike *K*-means where the number of clusters must be set *a-priori*, in MAP-DP, a specific parameter (the prior count) controls the rate of creation of new clusters. Hence, by a small increment in algorithmic complexity, we obtain a major increase in clustering performance and applicability, making MAP-DP a useful clustering tool for a wider range of applications than *K*-means.

MAP-DP is motivated by the need for more flexible and principled clustering techniques, that at the same time are easy to interpret, while being computationally and technically affordable for a wide range of problems and users. With recent rapid advancements in probabilistic modeling, the gap between technically sophisticated but complex models and simple yet scalable inference approaches that are usable in practice, is increasing. This is why in this work, we posit a flexible probabilistic model, yet pursue inference in that model using a straightforward algorithm that is easy to implement and interpret.

The generality and the simplicity of our principled, MAP-based approach makes it reasonable to adapt to many other flexible structures, that have, so far, found little practical use because of the computational complexity of their inference algorithms. Some BNP models that are somewhat related to the DP but add additional flexibility are the *Pitman-Yor process* which generalizes the CRP [42] resulting in a similar infinite mixture model but with faster cluster growth; *hierarchical DPs* [43], a principled framework for multilevel clustering; *infinite Hidden Markov models* [44] that give us machinery for clustering time-dependent data without fixing the number of states a priori; and *Indian buffet processes* [45] that underpin *infinite latent feature* models, which are used to model clustering problems where observations are allowed to be assigned to multiple groups.

## Appendix

### A Implementation practicalities

As with all algorithms, implementation details can matter in practice. We discuss a few observations here:

- *Empty clusters*. In MAP-DP, as with *K*-means, it is always possible that a cluster ceases to have any data points assigned to it. In that case, since $N_{k}^{-i}=0$, then it will be impossible in future iterations for data points to be assigned to that cluster label. So, it is reasonable to drop that label and re-assign the remaining non-empty clusters because the additional empty clusters are merely a wasted computational overhead. The MAP-DP algorithm (Algorithm 3) can be readily modified to do this; the most sensible place to do this is immediately after lines 12 or 13.
- *Dominating reinforcement* *on initialization*. Collapsing out the cluster parameters causes the cluster geometry to be very robust, for example, largely insensitive to outliers. However, there is an unwanted side-effect of this robustness: because MAP-DP (Algorithm 3) is initialized with one single large cluster, the reinforcement (rich-get-richer) effect of the DP can dominate over the geometry to cause MAP-DP to become trapped in the undesirable configuration where no new clusters can be generated. (Note that this is a problem for Gibbs sampling as well, but in theory at least, Gibbs can escape local minima after sufficient iterations, whereas MAP-DP cannot). Overcoming this reinforcement requires a prior count *N*_0_ on the order of the magnitude of *N*, but this would usually create many spurious small clusters. To avoid this side-effect, a practical solution removes the reinforcement effect due to this particular initialization scheme by inserting $N_{1}^{-i}=1$ in between lines 9 and 10 (Algorithm 3), only on the first iteration.
- *Numerical computation of negative log likelihood*. Computing the NLL (Algorithm 3 line 13) requires evaluating ln *Γ*(*N*_*k*_) terms which are difficult to estimate with high precision for large values of *N*_*k*_. As a result the NLL can develop small numerical errors which can cause the NLL to increase slightly over iterations. A simple practical fix is to replace the convergence test with absolute values, i.e. |*E*_old_ − *E*_new_| < *ϵ* in line 14.

### B Randomized restarts

As MAP-DP is a completely deterministic algorithm, if applied to the same data set with the same choice of input parameters, it will always produce the same clustering result. However, since the algorithm is not guaranteed to find the global maximum of the likelihood Eq (11), it is important to attempt to restart the algorithm from different initial conditions to gain confidence that the MAP-DP clustering solution is a good one. Since there are no random quantities at the start of the MAP-DP algorithm, one viable approach is to perform a random permutation of the order in which the data points are visited by the algorithm. The quantity *E* Eq (12) at convergence can be compared across many random permutations of the ordering of the data, and the clustering partition with the lowest *E* chosen as the best estimate.

### C Obtaining cluster centroids

As explained in the introduction, MAP-DP does not explicitly compute estimates of the cluster centroids, but this is easy to do after convergence if required. The cluster posterior hyper parameters *θ*_*k*_ can be estimated using the appropriate Bayesian updating formulae for each data type, given in (S1 Material). For example, for spherical normal data with known variance:

$$\begin{array}{ccc} \sigma_{k} & = & {\left(\frac{1}{\sigma_{0}^{2}}+\frac{1}{{\hat{\sigma }}^{2}}N_{k}\right)}^{-1}\\ \mu_{k} & = & \sigma_{k}\left(\frac{\mu_{0}}{\sigma_{0}^{2}}+\frac{1}{{\hat{\sigma }}^{2}}\sum_{i:z_{i}=k}x_{i}\right) \end{array}$$ (14)

Using these parameters, useful properties of the posterior predictive distribution *f*(*x*|*θ*_*k*_) can be computed, for example, in the case of spherical normal data, the posterior predictive distribution is itself normal, with mode *μ*_*k*_. Indeed, this quantity plays an analogous role to the cluster means estimated using *K*-means.

### D Out-of-sample predictions

To make out-of-sample predictions we suggest two approaches to compute the out-of-sample likelihood for a new observation *x*_*N*+1_, approaches which differ in the way the indicator *z*_*N*+1_ is estimated.

1. *Mixture predictive density*. The unknown indicator *z*_*N*+1_ can be integrated out resulting in a mixture density:
  $$\begin{array}{c} p\left(x_{N+1}|N_{0},z,X\right)=\sum_{k=1}^{K+1}p\left(z_{N+1}=k|N_{0},z,X\right)p\left(x_{N+1}|z,X,z_{N+1}=k\right) \end{array}$$ (15)
  The assignment probability *p*(*z*_*N*+1_ = *k*|*z*_*N*_, *N*_0_) is $\frac{N_{k}}{N_{0}+N}$ for an existing cluster and $\frac{N_{0}}{N_{0}+N}$ for a new cluster. The second term corresponds to the predictive distribution of *N* + 1 point $p(x_{N+1}|z,X,z_{N+1}=k)=f(x_{N+1}|\theta_{k}^{-(N+1)})$.
2. *MAP predictive density*. We can also use a point estimate for *z*_*N*+1_ by picking the minimum negative log posterior of the indicator *p*(*z*_*N*+1_|*x*_*N*+1_, *N*_0_) or equivalently:
  $$\begin{array}{c} z_{N+1}^{\text{MAP}}=\underset{k\in 1,\ldots ,K,K+1}{arg min}\left[-\text{ln }p\left(x_{N+1}|z,X,z_{N+1}=k\right)-\text{ln }p\left(z_{N+1}=k|N_{0},z,X\right)\right] \end{array}$$ (16)
  where *p*(*x*_*N*+1_|*z*, *X*, *z*_*N*+1_ = *k*) and *p*(*z*_*N*+1_ = *k*|*N*_0_, *z*, *X*) are computed as in the approach above. Once we have evaluated the MAP assignment for point *N* + 1, $z_{N+1}^{\text{MAP}}=k^{*}$ we model *x*_*N*+1_ with predictive density $p(x_{N+1}|z,X,z_{N+1}^{MAP}=k^{*})=f(x_{N+1}|\theta_{k^{*}}^{-(N+1)})$.

The first (marginalization) approach is used in Blei and Jordan [15] and is more robust as it incorporates the probability mass of all cluster components while the second (modal) approach can be useful in cases where only a point prediction is needed.

### E Missing data

In MAP-DP, we can learn missing data as a natural extension of the algorithm due to its derivation from Gibbs sampling: MAP-DP can be seen as a simplification of Gibbs sampling where the sampling step is replaced with maximization. The Gibbs sampler provides us with a general, consistent and natural way of learning missing values in the data without making further assumptions, as a part of the learning algorithm. That is, we can treat the missing values from the data as latent variables and sample them iteratively from the corresponding posterior one at a time, holding the other random quantities fixed. In this framework, Gibbs sampling remains consistent as its convergence on the target distribution is still ensured. (Note that this approach is related to the ignorability assumption of Rubin [46] where the missingness mechanism can be safely ignored in the modeling. Molenberghs et al. [47] have shown that more complex models which model the missingness mechanism cannot be distinguished from the ignorable model on an empirical basis.)

Coming from that end, we suggest the MAP equivalent of that approach. We treat the missing values from the data set as latent variables and so update them by maximizing the corresponding posterior distribution one at a time, holding the other unknown quantities fixed. In MAP-DP, the only random quantity is the cluster indicators *z*_1_, …, *z*_*N*_ and we learn those with the iterative MAP procedure given the observations *x*_1_, …, *x*_*N*_. Consider some of the variables of the *M*-dimensional *x*_1_, …, *x*_*N*_ are missing, then we will denote the vectors of missing values from each observations as $x_{1}^{*},\ldots ,x_{N}^{*}$ with $x_{i}^{*}={\left(x_{i,m}^{*}\right)}_{m=1}^{M}$ where $x_{i,m}^{*}$ is empty if feature *m* of the observation *x*_*i*_ has been observed. MAP-DP for missing data proceeds as follows:

1. For each feature *m* = 1, …, *M*, sample all of the missing values $x_{1,m}^{*},\ldots ,x_{N,m}^{*}$ from the likelihood for that variable given the prior parameters *f*(*x*_*i*_|*θ*_0,*m*_). Note that we assume independent priors and that the likelihood for the different variables can take different forms, as in the case study 6.
2. Combine the sampled missing variables with the observed ones and proceed to update the cluster indicators *z*_1_, …, *z*_*N*_, treating all of the variables as known. The indicators *z*_1_, …, *z*_*N*_ are updated as above, by computing for each point *i*, the *K* + 1 quantities *d*_*i*,1_, …, *d*_*iK*_, *d*_*i*,*K*+1_ and computing $z_{i}=\arg\min_{k\in 1,\ldots ,,K+1}\;[d_{i,k}-\text{ln }N_{k}^{-i}]$.
3. Once all of the indicators *z*_1_, …, *z*_*N*_ are updated, update the missing variables $x_{1}^{*},\ldots ,x_{N}^{*}$. For each point *i*, update $x_{i}^{*}$ by taking the mode of the corresponding likelihood $x_{i,d}^{*}={arg max}_{x_{\cdot ,d}}\;f(x_{\cdot ,d}|\theta_{z_{i}}^{-i})$. For the elliptical model we can take the mode of each dimension independently $x_{i,d}^{*}={arg max}_{x_{\cdot ,d}}\;f(x_{\cdot ,d}|\theta_{z_{i},d}^{-i})$. After all $x_{1}^{*},\ldots ,x_{N}^{*}$ are updated, go back to step 2 and update the cluster indicators *z*_1_, … *z*_*N*_, now using the observations and the updated missing variables.

### F Estimating the model hyper parameters (*θ*_0_, *N*_0_)

In Bayesian models, ideally we would like to choose our hyper parameters (*θ*_0_, *N*_0_) from some additional information that we have for the data. This could be related to the way data is collected, the nature of the data or expert knowledge about the particular problem at hand. For instance when there is prior knowledge about the expected number of clusters, the relation *E*[*K*^+^] = *N*_0_ log *N* could be used to set *N*_0_.

In cases where this is not feasible, we have considered the following alternatives:

1. *Empirical Bayes* (EB). Set the hyper parameters to their corresponding maximum marginal likelihood values. The maximum marginal likelihood expression for *θ*_0_ will be different for the different data types and will not always be available in closed form. Usually they can be obtained from the parameter updates in (S1 Material) by omitting the prior terms. In MAP-DP, the maximum likelihood estimates for the hyper parameters *θ*_0_ coincide with EB estimates as the cluster parameters *θ* have already been integrated out. In fact, in the simple case of conjugate exponential family models, the EB estimates and the maximum likelihood estimates for the model hyper parameters are quite similar. That is why it is common to use the maximum likelihood estimates as a simple approximation to the EB estimate. This approach is referred to as *parametric EB point estimation* [48]. Note that using EB to learn the hyper parameter *N*_0_ would not be efficient because there is no closed form expression for the marginal likelihood (see point 3 below, and Eq (17).
2. *Multiple restarts*. Run MAP-DP with different starting values for each of the hyper parameters (*θ*_0_, *N*_0_), compute the NLL from Eq (12) including the *C*(*N*_0_, *N*) term at convergence, change one of the hyper parameters holding the rest fixed and then restart MAP-DP with the prior parameter. Set that hyper parameter to the value resulting in smallest NLL and proceed in the same way for the next hyper parameter of the model. *Bayesian optimisation* [49] has also been proposed to fit model hyper parameters but requires the specification of a Gaussian Process and associated priors that may be challenging in practice. We have therefore not utilised this approach and prefer the simpler greedy search approach. However in certain cases BO may be more efficient in terms of the number of MAP-DP runs required.
3. *MAP estimate*. Place a prior on the hyper parameter of interest and numerically compute the mode of the posterior. For instance, by using a gamma prior on *N*_0_, *p*(*N*_0_) = Gamma(*a*_*N*_0__, *b*_*N*_0__), the posterior is proportional to:
  $$\begin{array}{c} p\left(N_{0}|N,K\right)\propto \frac{\Gamma \left(N_{0}\right)}{\Gamma \left(N_{0}+N\right)}N_{0}^{K+a_{N_{0}}-1}\exp\left[-b_{N_{0}}N_{0}\right] \end{array}$$ (17)
  We can numerically minimize the negative log of this posterior using e.g. Newton’s method. To ensure the solution is positive we can compute the gradient with respect to ln *N*_0_: as Rasmussen [50] notes *p*(ln *N*_0_|*N*, *K*^+^) is log-concave and therefore has a unique maximum.
4. *Cross-validation*. By considering a finite set of values for (*θ*_0_, *N*_0_), choose the value corresponding to the minimum, average, out-of-sample likelihood across all cross-validation repetitions (see Appendix D). This approach is taken in Blei and Jordan [15] to compare different inference methods.

We have found the second approach to be the most effective where empirical Bayes can be used to obtain the values of the hyper parameters at the first run of MAP-DP. For small datasets we recommend using the cross-validation approach as it can be less prone to overfitting.

## Supporting Information

S1 Function This is an example function in MATLAB implementing MAP-DP algorithm for Gaussian data with unknown mean and precision. (M)

S1 Script This is a script evaluating the S1 Function on synthetic data. (M)

S1 Material We include detailed expressions for how to update cluster hyper parameters and other probabilities whenever the analyzed data type is changed. (PDF)
