# Supplementary material for: What to Do When K-Means Clustering Fails: A Simple yet Principled Alternative Algorithm
Source: PLoS One. 2016 Sep 26;11(9):e0162259. doi: 10.1371/journal.pone.0162259 (PMC5036949; doi:10.1371/journal.pone.0162259)
Supplement: S1 Material — (PDF) [file pone.0162259.s003.pdf]

Updating cluster hyper parameters and computing negative log predictive distribution functions

In the generalized MAP-DP algorithm (Algorithm 3), the computation of the variables $d_{i,k}$ and $d_{i,K+1}$ (algorithm lines 8,9) requires the collapsed prior predictive distribution $f(x|\theta_0)$, and also the collapsed posterior predictive distribution $f\left(x|\theta_k^{-i}\right)$. This predictive distribution requires the updated cluster posterior hyper parameters θ_k^{-i} (algorithm line 7). These updates depend upon the distribution, and the data type, of each data point x_i. When the distribution is from the *exponential family*, the prior distribution over the parameters can be chosen to be *conjugate*: the prior over the parameters of the data distribution and the posterior have the same form of distribution. This simplifies the hyper parameter updates, and, furthermore, the form of the prior and posterior predictive distributions is the same and is available in closed form. The table below lists some possible data types and distributions, their conjugate prior/posterior distribution, the names given to the hyper parameters and the corresponding name of the predictive distributions. We discuss each case in more detail in the subsequent sections.

Distribution of data x_i	Data type	Conjugate prior/posterior	Hyper parameters θ	Predictive distribution
Spherical normal (known variance)	$x \in \mathbb{R}^D$	Spherical normal	$\left(\mu, \sigma^2\right)$	Spherical normal
Multivariate normal (known covariance)	$x \in \mathbb{R}^D$	Multivariate normal	(μ, Σ)	Multivariate normal
Multivariate normal	$x \in \mathbb{R}^D$	Normal-Wishart	(m, c, B, a)	Multivariate Student-t
Exponential	$x \in \mathbb{R}$, $x \geq 0$	Gamma	(α, β)	Lomax
Categorical	$x \in \{1, 2, \ldots D\}$	Dirichlet	$(\alpha_1, \ldots, \alpha_D)$	Dirichlet-multinomial
Binomial	$x \in \{0, 1, \ldots n\}$	Beta	(α, β)	Beta-binomial
Poisson	$x \in \mathbb{Z}$, $x \geq 0$	Gamma	(α, β)	Negative-binomial
Geometric	$x \in \mathbb{Z}$, $x \geq 0$	Beta	(α, β)	Ratio of beta functions

Spherical normal data with known variance

This is the variant of MAP-DP described in Algorithm 2. When each data point $x \in \mathbb{R}^D$ is assumed to be spherical Gaussian with known variance $\hat{\sigma}^2$ shared across dimensions, the conjugate prior distribution of the Gaussian mean vector parameter $\mu \in \mathbb{R}^D$ is also spherical normal with hyper parameters $\theta_0 = \left(\mu_0, \sigma_0^2\right)$. Then the posterior distribution for each cluster is also spherical normal with hyper parameters $\theta_k^{-i} = \left(\mu_k^{-i}, \sigma_k^{-i}\right)$. The hyper parameter updates (Algorithm 3, line 7) for each cluster are:

$$
\begin{aligned}
\sigma_k^{-i} &= \left(\frac{1}{\sigma_0^2} + \frac{1}{\hat{\sigma}^2} N_k^{-i}\right)^{-1} \\
\mu_k^{-i} &= \sigma_k^{-i} \left(\frac{\mu_0}{\sigma_0^2} + \frac{1}{\hat{\sigma}^2} \sum_{j:z_j=k, j\neq i} x_j\right)
\end{aligned}
\tag{1}
$$

The predictive distributions $f\left(x|\theta_0\right)$ and $f\left(x\left|\theta_k^{-i}\right.\right)$ are D-dimensional spherical normal distributions, whose negative logs are:

$$
-\ln f\left(x|\theta\right) = \frac{1}{2\left(\sigma^2 + \hat{\sigma}^2\right)} \left\|x - \mu\right\|_2^2 + \frac{D}{2} \ln\left(\sigma^2 + \hat{\sigma}^2\right) + \frac{D}{2} \ln\left(2\pi\right)
\tag{2}
$$

Note that since the normalization term $\frac{D}{2}\ln\left(2\pi\right)$ is common to both predictive distributions, it can be omitted when computing $d_{i,k}$ and $d_{i,K+1}$ in the algorithm.

Multivariate normal data with known covariance

For data points $x \in \mathbb{R}^D$ assumed to be multivariate Gaussian with known covariance matrix $\hat{\Sigma}$, the conjugate prior distribution of the Gaussian mean vector parameter is also multivariate normal with hyper parameters $\theta_0 = \left(\mu_0, \Sigma_0\right)$. The posterior distribution for each cluster is also multivariate normal with hyper parameters $\theta_k^{-i} = \left(\mu_k^{-i}, \Sigma_k^{-i}\right)$. The hyper parameter updates are:

$$
\begin{aligned}
\Sigma_k^{-i} &= \left(\Sigma_0^{-1} + \hat{\Sigma}^{-1} N_k^{-i}\right)^{-1} \\
\mu_k^{-i} &= \Sigma_k^{-i} \left(\Sigma_0^{-1}\mu_0 + \hat{\Sigma}^{-1} \sum_{j:z_j=k, j\neq i} x_j\right)
\end{aligned}
\tag{3}
$$

The predictive distributions $f\left(x|\theta_0\right)$ and $f\left(x\left|\theta_k^{-i}\right.\right)$ are D-dimensional normal distributions, whose negative logs are:

$$
-\ln f\left(x|\theta\right) = \tfrac{1}{2}\left(x-\mu\right)^T \left(\Sigma + \hat{\Sigma}\right)^{-1} \left(x - \mu\right) + \frac{D}{2} \ln\left|\Sigma + \hat{\Sigma}\right| + \frac{D}{2} \ln\left(2\pi\right)
\tag{4}
$$

Since the normalization term $\frac{D}{2}\ln\left(2\pi\right)$ is common to both predictive distributions, it can be omitted when computing $d_{i,k}$ and $d_{i,K+1}$ in the algorithm.

Multivariate Gaussian data

When each data point $x \in \mathbb{R}^D$ is assumed to be multivariate Gaussian with unknown mean vector and covariance matrix, the conjugate prior distribution of the Gaussian parameters is Normal-Wishart, with hyper parameters $\theta_0 = \left(m_0, c_0, B_0, a_0\right)$. Then, the posterior distribution for each cluster is also Normal-Wishart, with hyper parameters $\theta_k^{-i} = \left(m_k^{-i}, c_k^{-i}, B_k^{-i}, a_k^{-i}\right)$. These are updated for each cluster according to:

$$\begin{aligned}
m_k^{-i} =& \frac{c_0 m_0 + N_k^{-i} \bar{x}_k^{-i}}{c_0 + N_k^{-i}} \\
c_k^{-i} =& c_0 + N_k^{-i} \\
B_k^{-i} =& \left(B_0^{-1} + S_k^{-i} + \frac{c_0 N_k^{-i}}{c_0 + N_k^{-i}} \left(\bar{x}_k^{-i} - m_0 \right) \left(\bar{x}_k^{-i} - m_0 \right)^T \right)^{-1} \\
a_k^{-i} =& a_0 + N_k^{-i}
\end{aligned} \tag{5}$$

where:

$$\begin{aligned}
\bar{x}_k^{-i} &= \frac{1}{N_k^{-i}} \sum_{j:z_j=k, j\neq i} x_j \\
S_k^{-i} &= \sum_{j:z_j=k, j\neq i} \left(x_i - \bar{x}_k^{-i} \right) \left(x_i - \bar{x}_k^{-i} \right)^T
\end{aligned} \tag{6}$$

The predictive distributions $f\left(x|\theta_0\right)$ and $f\left(x\left|\theta_k^{-i}\right.\right)$ are D-dimensional multivariate Student-t distributions, whose negative log, written in terms of the parameters (μ, Λ, ν) is:

$$\begin{aligned}
-\ln f\left(x|\theta\right) &= \frac{\nu + D}{2} \ln \left[1 + \nu^{-1} \left(x - \mu \right)^T \Lambda \left(x - \mu \right) \right] - \frac{1}{2} \ln |\Lambda| + \ln \Gamma \left(\frac{\nu}{2} \right) \\
&+ \frac{D}{2} \ln \left(\nu \pi \right) - \ln \Gamma \left(\frac{\nu + D}{2} \right)
\end{aligned} \tag{7}$$

where the Student-t parameters (μ, Λ, ν) are given in terms of the Normal-Wishart parameters $\mu = m$, $\nu = a - D + 1$ and $\Lambda = \frac{c\nu}{c+1} B$. We note that fast incremental updates of all these parameters are possible when including and then removing a single data point from a cluster, see Raykov et al. [1] for further details.

Exponential data

Given data points $x \in \mathbb{R}$, $x \geq 0$ assumed to be exponentially-distributed, the conjugate prior over the exponential rate parameter is the gamma distribution. This gamma distribution has hyper parameters $\theta_0 = (\alpha, \beta)$ (shape, rate). So, the posterior probability of the rate parameter is also gamma, and the cluster hyper parameter $\theta_k^{-i} = \left(\alpha_k^{-i}, \beta_k^{-i} \right)$ are updated using:

$$\begin{aligned}
\alpha_k^{-i} &= \alpha_0 + \sum_{j:z_j=k, j\neq i} x_j \\
\beta_k^{-i} &= \beta_0 + N_k^{-i}
\end{aligned} \tag{8}$$

The predictive distributions $f\left(x|\theta_0\right)$ and $f\left(x\left|\theta_k^{-i}\right.\right)$ are the so-called *Lomax* distribution, with negative log:

$$-\ln f\left(x|\theta\right) = -\ln \alpha - \alpha \ln \beta + (\alpha + 1) \ln \left(x + \beta \right) \tag{9}$$

Categorical data

For categorical data which can take on one of $D > 1$ possible values, $x \in \{1, 2, \ldots D\}$, the conjugate prior over the D outcome probability parameters of this distribution are Dirichlet distributed. This Dirichlet distribution has hyper parameters

$\theta_0 = (\alpha_{0,1}, \ldots, \alpha_{0,D})$. So, the posterior outcome probability parameters for each cluster are also Dirichlet, and for each cluster the D entries in the cluster hyper parameter $\theta_k^{-i} = \alpha_k^{-i}$ are updated using:

$$\alpha_{k,d}^{-i} = \alpha_{0,d} + \sum_{j:z_j=k,j\neq i} \delta\left(x_j, d\right) \text{ for } d = 1, \ldots, D \tag{10}$$

where $\delta\left(x, y\right) = 1$ if $x = y$ and 0 otherwise. The predictive distributions $f\left(x|\theta_0\right)$ and $f\left(x\left|\theta_k^{-i}\right.\right)$ are special cases of the Dirichlet-multinomial distribution, with negative log:

$$-\ln f\left(x|\theta\right) = -\ln \alpha_x + \ln \sum_{d=1}^{D} \alpha_d \tag{11}$$

Binomial data

In the case of binomial data where the data can take on $x \in \{0, 1, \ldots n\}$ for $n > 0$, the conjugate prior over the binomial success probability parameter is beta distributed, with hyper parameters $\theta_0 = (\alpha_0, \beta_0)$. By conjugacy, the posterior cluster parameters are also beta distributed with hyper parameters $\theta_k^{-i} = \left(\alpha_k^{-i}, \beta_k^{-i}\right)$, and are updated according to:

$$\begin{aligned} \alpha_k^{-i} &= \alpha_0 + \sum_{j:z_j=k,j\neq i} x_j \\ \beta_k^{-i} &= \beta_0 + N_k^{-i} n - \sum_{j:z_j=k,j\neq i} x_j \end{aligned} \tag{12}$$

For such binomial data, the predictive distributions $f\left(x|\theta_0\right)$ and $f\left(x\left|\theta_k^{-i}\right.\right)$ are beta-binomial, with negative log:

$$-\ln f\left(x|\theta\right) = -\ln \begin{pmatrix} n \\ x \end{pmatrix} - \ln B\left(x+\alpha, n-x+\beta\right) + \ln B\left(\alpha, \beta\right) \tag{13}$$

where $B\left(\cdot,\cdot\right)$ is the beta function:

$$B\left(\alpha, \beta\right) = \frac{\Gamma\left(\alpha\right)\Gamma\left(\beta\right)}{\Gamma\left(\alpha+\beta\right)} \tag{14}$$

Poisson data

For positive integer Poisson count data $x \in \mathbb{Z}$, $x \geq 0$, the conjugate prior over the single rate parameter is the gamma distribution with hyper parameters $\theta_0 = (\alpha_0, \beta_0)$ (shape and rate, respectively). The posterior cluster parameters are similarly gamma distributed with hyper parameters $\theta_k^{-i} = \left(\alpha_k^{-i}, \beta_k^{-i}\right)$. The updates for these hyper parameters are:

$$\begin{aligned} \alpha_k^{-i} &= \alpha_0 + \sum_{j:z_j=k,j\neq i} x_j \\ \beta_k^{-i} &= \beta_0 + N_k^{-i} \end{aligned} \tag{15}$$

For Poisson count data, the predictive distributions $f\left(x|\theta_0\right)$ and $f\left(x\left|\theta_k^{-i}\right.\right)$ are negative binomial distributed with negative log:

$$-\ln f\left(x|\theta\right) = -\ln \begin{pmatrix} \alpha+\beta-1 \\ \beta \end{pmatrix} - \alpha \ln\left(1-x\right) - \beta \ln x \tag{16}$$

Geometric data

In the case of positive integer data $x \in \mathbb{Z}$, $x \geq 0$ which is assumed to be geometrically-distributed, the conjugate prior over the single success probability parameter is the beta distribution with hyper parameters $\theta_0 = (\alpha_0, \beta_0)$. The posterior cluster parameters are similarly beta distributed with hyper parameters $\theta_k^{-i} = \left(\alpha_k^{-i}, \beta_k^{-i}\right)$. The updates for these hyper parameters are:

$$\begin{aligned} \alpha_k^{-i} &= \alpha_0 + N_k^{-i} \\ \beta_k^{-i} &= \beta_0 + \sum_{j:z_j=k, j\neq i} x_j \end{aligned} \tag{17}$$

For geometric data, the predictive distributions $f(x|\theta_0)$ and $f\left(x\left|\theta_k^{-i}\right.\right)$ have negative log:

$$-\ln f(x|\theta) = -\ln B(\alpha+1, \beta+x) + \ln B(\alpha, \beta) \tag{18}$$

where $B(\cdot,\cdot)$ is the beta function described above.

References

1. Raykov YP, Boukouvalas A, Little MA. Simple approximate MAP Inference for Dirichlet processes. Aston University; 2014. arXiv:1411.0939.
